# Case report: Identification of the tropical rat mite (*Ornithonyssus bacoti*) on a domestic donkey in France

**DOI:** 10.3389/fvets.2023.1141290

**Published:** 2023-05-26

**Authors:** Mirabela Oana Dumitrache, Adriana Györke, Florie Julien, Jevgenija Kondratjeva, Marie-Christine Cadiergues

**Affiliations:** ^1^Department of Parasitology and Parasitic Diseases, Faculty of Veterinary Medicine, University of Agricultural Science and Veterinary Medicine Cluj-Napoca, Cluj-Napoca, Romania; ^2^Department of Clinical Sciences, Université de Toulouse, ENVT, Toulouse, France; ^3^Selarl Hippovet Aude, Laure Minervois, France; ^4^INFINITy, Université de Toulouse, Inserm, CNRS, UPS, Toulouse, France

**Keywords:** *Ornithonyssus bacoti*, tropical rat mite, donkey, parasites, molecular diagnosis

## Abstract

A 25-year-old donkey was referred for a generalized, pruritic and severe exfoliative dermatitis that had been evolving for several years, with deterioration in the last few months. Close examination of the skin surface revealed numerous small, dark, mobile elements identified as *Ornithonyssus bacoti* confirmed by DNA sequencing. The severity, type and topography of the lesions called for complementary examinations, leading to a second diagnosis of cutaneous epitheliotropic T-cell lymphoma. The lack of clinical improvement after antiparasitic therapy despite parasite clearance, suggests opportunistic behavior of *Ornithonyssus bacoti*. To the best of our knowledge, this is the first report of the presence of a tropical rat mite on a donkey, thus expanding the known host spectrum of this zoonotic parasite. Further potential questions concern the implication of this new host as a possible source of human contamination.

## 1. Introduction

*Ornithonyssus bacoti* also known as “the tropical rat mite” is a zoonotic hematophagous mesostigmatid mite belonging to the family Macronyssidae that parasites preferentially wild rodents (1). Although infestations may remain clinically unnoticed, severe outbreaks with major dermatologic and systemic impairment have been reported in the rodent population. Moreover, the mite has been implicated as the etiological agent of human pruritic dermatitis on several occasions (2, 3). Pet rodents are traditionally considered as the source of this epizoonosis and the close contact between the animal and human body appears to facilitate transmission (2). *Ornithonyssus bacoti* is an obligate intermittent hematophagous non-borrowing mite. The relatively large size of the parasite, 0.5–1.5 mm, means it is visible to the naked eye. It displays the typical behavior of a nest parasite, and is rarely found on its host except when feeding, which mainly happens at night. During the non-feeding period, it returns to its habitat close to the host (2, 3). Infestations with *O. bacoti* can occur in temperate and tropical climates worldwide. The preferred natural hosts are wild rats (*Rattus norvegicus, Rattus rattus*) and mice (*Mus musculus*) (3), but tropical rat mites have also been reported on other hosts such as gerbil (*Meriones unguiculatus*), hamster (*Mesocricetus auratus*), red-bellied squirrel (*Callosciurus erythraeus*) and other small mammals (pet rabbits, guinea pigs, hedgehogs, and sugar gliders) (4–7). Outbreaks of *O. bacoti* in rodent facilities of different research institutions have been reported on several occasions (1, 8, 9). *Ornithonyssus bacoti* has been demonstrated to harbor many zoonotic agents such as *Bartonella* spp., *Coxiella burnetii, Borrelia* spp., *Rickettsia* spp., and hantavirus (10, 11).

The current report describes an opportunistic infestation with *O. bacoti*, confirmed by molecular methods, in a donkey with epitheliotropic T-cell lymphoma.

## 2. Case presentation

**Figure 1:**
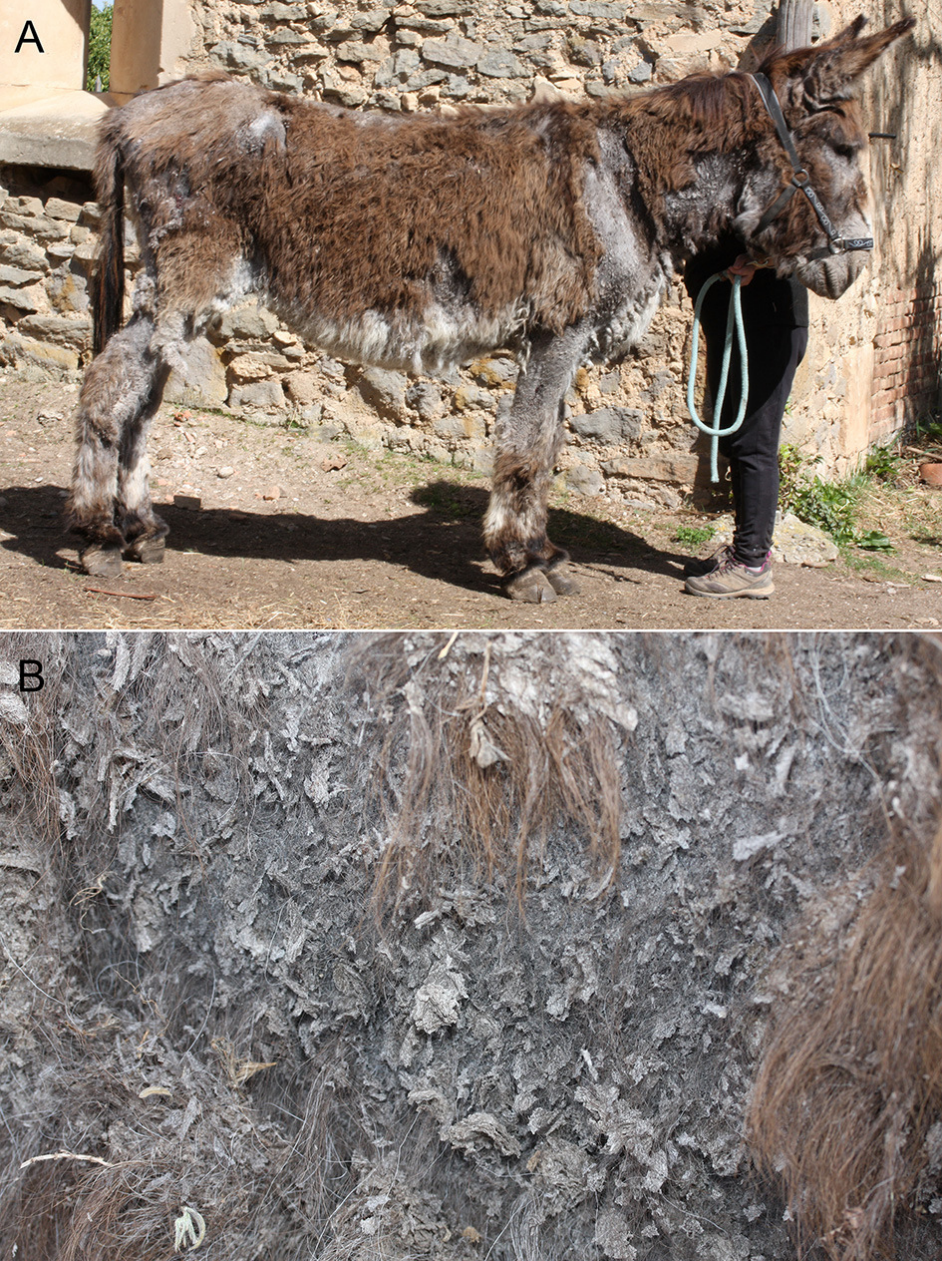
**(A)** General view showing severe, extended exfoliative dermatitis with alopecia and scaling. **(B)** Close view showing very large, thin grayish scales in alopecic areas.

A 25-year-old donkey gelding was referred due to a 7–8-year history of exfoliative dermatitis associated with mild to moderate pruritus. The clinical manifestations remained fairly stable since the beginning but deteriorated slightly in recent months. The owner, a private breeder, reported progressive weight loss, but no other systemic signs. For the last 10 years, the donkey had lived in a 250 m^2^ enclosed field with a 10 m^2^ shelter located in the South of France. The donkey shared the field with two other donkeys and occasionally with horses originating from the same area, none of which ever displayed any skin problems. The presence of poultry nearby was reported, but with no possibility of direct contact with the equines. A water canal was located in the close proximity of the field, where rodents were occasionally observed as well as biting insects in summer. However, no invasion of rodents was reported in the enclosure where the donkey was kept. The only treatment was occasional application of povidone iodine, without any improvement. The clinical examination, conducted outside in natural light, revealed a mildly underweight (body condition score 2/5), but otherwise frisky donkey. The dermatological lesions were extensive areas of alopecia, with numerous large, thin, grayish scales (Figure 1). Occasional erosions covered by a sero-hemorrhagic exudate were visible. The lesions extended to all mucocutaneous junctions. Close examination of scaly lesions revealed numerous dark, mobile elements about 1 mm in diameter. Following this examination, and despite the fact that they were wearing clinical gowns, protecting most of their bodies, and examination gloves, the investigators developed transient, mild to moderate pruritus on their arms, trunk, and neck, without any noticeable lesions, that disappeared within 1 h. Despite careful and prolonged examination of the skin of the two in-contact donkeys, no similar specimens were found on them. Considering the history and the clinical and dermatological examinations, the differential diagnoses included ectoparasitic diseases, dermatophytosis, autoimmune diseases (i.e., pemphigus foliaceus), tumoral conditions (i.e., sarcoidosis, epitheliotropic lymphoma), or keratinisation disorders (i.e., primary seborrhoea, zinc-responsive dermatosis).

**Figure 2:**
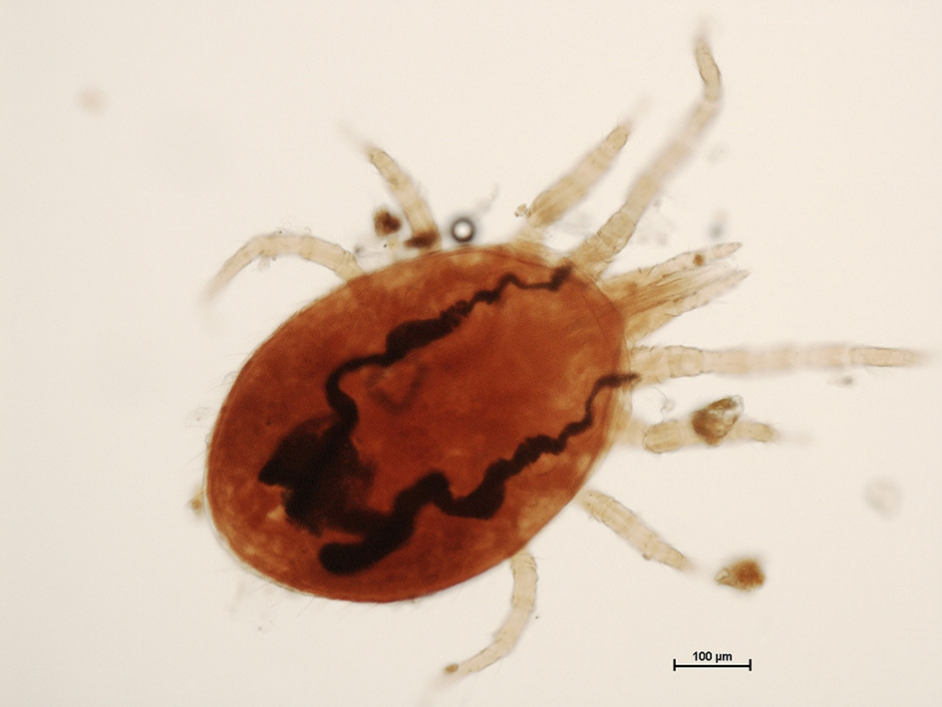
Adult tropical rat mite (*Ornithonyssus bacoti*) (Scale bar = 100 μm).

Mobile specimens and scaly material were collected from the donkey and examined microscopically, enabling the identification of mites at various developmental stages [adults (Figure 2), nymphs, larvae and eggs]. Initially, they were thought to be *Dermanyssus gallinae* (12). In order to confirm the morphological identification, genomic DNA was extracted from specimens (*n* = 3) collected from the donkey using a commercial kit (Isolate II Genomic DNA Kit, Bioline, London, UK) following the manufacturer's instructions. The COI gene fragment of 710-bp was PCR-amplified using primer pairs LCO1490/HC02198 (13). The PCR was performed in C1000TM Thermal Cycle (Bio-Rad Laboratories, Inc.) using 2x Green PCR Master Mix (Rovalab) in a final reaction volume of 25 μL. The amplification program consisted in an initial denaturation cycle at 95°C for 15 min, followed by 45 denaturation cycles at 94°C for 45 s, annealing at 47°C for 45 s, extension at 72°C for 90 s, and a final extension cycle at 72°C for 10 min. *Dermanyssus gallinae* specimens were used as positive controls during DNA extraction and PCR amplification. Purified water was used as negative control in PCR. PCR amplicons were visualized by electrophoresis in 1.5% agarose gel stained with SYBR Safe DNA gel stain (Invitrogen, Waltham, MA, USA). Gel Doc XR + Gel Documentation System (Bio-Rad) was used to visualize specific fragments. The length of the amplified DNA fragments was compared to the 100 bp genetic markers (O'GeneRuler 100 bp DNA Ladder, ready-to-use, Thermo Scientific). The PCR product was purified using FavorPrep GEL/PCR Purification Mini Kit (Favorgen Biotech Corp., Taiwan) and further sequenced (Macrogen Europe). The obtained sequences were compared with the available sequence in GenBank. PCR amplification yielded a product of ~710 bp for both types of specimen (analyzed mites, and *D. gallinae*). BLAST analysis of the analyzed mites showed between 98.4 and 99.51% identity with *Ornithonyssus bacoti* of unknown (acc. no. FM179677.2) (14), and Chinese (acc. no. MH553336.1- MH553338.1) origin, respectively. The sequence of our specimen was submitted to GenBank under accession number OQ195774.

Following identification of the mites, deltamethrin (Butox 7.5% pour-on, MSD, Beaucouzé, France−10 mL) was topically applied twice at an interval of 2 weeks on all three donkeys. No intervention was made in the environment and the animals were left in their original enclosure. Microscope examination of control superficial skin scrapings, performed 3 weeks later, did not reveal any remaining mites. Starting from day 14, the investigators stopped developing pruritus following the examinations. However, despite parasite clearance, no signs of clinical improvement were observed. Further investigations (i.e., cytology, complete blood count and blood smear, and biochemistry, bacterial culture, fungal culture, histopathological examination of skin biopsy and molecular clonality PCR) were thus performed and confirmed the first report of cutaneous epitheliotropic T-cell lymphoma in a donkey (15).

## 3. Discussion

To the best of our knowledge, *O. bacoti* has not been previously reported on donkeys or on any other equids. The main natural source of contamination is wild rats and mice, while pet and laboratory rodents may also act as reservoirs (3). In the present case, the source of the mite is possibly wild rodents that were observed near the water canal located in the proximity of the field. Although the owner did not notice a rodent invasion in the enclosed field where the donkey was kept, this remains the main hypothesis regarding the origin of the parasites. Furthermore, visual assessment of the presence or absence of rodents in a given ecosystem is not an efficient method of estimating a rodent population. Monitoring remains a present challenge even when more documented and specific methods are used (16).

Infestation with *O. bacoti* is usually asymptomatic. However, in the case of severe infestation in rodents, symptoms such as pruritus, alopecia, erythema and anemia may occur (1). In the present case, the complete blood count and blood smear evaluation revealed mild normocytic normochromic anemia, which could be explained by the large number of parasites observed on the donkey's skin. However, the skin lesions could not be attributed to infestation by the parasite *O. bacoti*, since no clinical improvement was observed following the successful antiparasitic treatment. An interesting aspect is that none of the other animals housed temporarily or permanently in the same fenced field exhibited clinical signs compatible with *O. bacoti* infestation. Moreover, careful examination of the animals in contact with the donkey revealed no mites on the surface of their skin. In this context, we assume that pre-existing skin lesions due to the cutaneous epitheliotropic T-cell lymphoma were factors favoring infestation by the parasite. This finding suggests another context in which contamination with the tropical rat mite can occur, adding important data to the known ecology of this parasite. To date, increased skin contact with contaminated rodents and/or decreased availability of preferred hosts have been the main scenarios for contamination of non-preferred hosts (2, 7, 9). It is important to underline the need for searching this mite in symptomatic and asymptomatic animals as well as the necessity of antiparasitic treatments to prevent/control this infestation.

Among mesostigmatid mites, the Macronyssidae and Dermanyssidae families are most frequently associated with human pruritic dermatitis (3). In the present case, handling the contaminated animal led to transient itching in the people who conducted the examination. Moreover, the species best known for their implication in public health, *O. bacoti*, the tropical rat mite, *O. sylviarum*, the Nordic bird mite and *D. gallinae*, the red bird mite (17), share morphological characteristics, and are often confused (2). This easily explains our initial misdiagnosis of *D. gallinae* following microscope examination of the mites. Hence, to implement efficient control measures, efforts should first focus on species identification. What is more, in the case of zoonotic parasites, it is essential to also correctly identify the source of the parasite. Accurate species identification, either by morphological features or using molecular techniques, will facilitate the identification of potential animal reservoirs.

The present case report is the first evidence of *O. bacoti* on donkey suggesting that the presence of severe exfoliative skin lesions favors the parasitic infestation. Like many other parasites, *O. bacoti* displays highly adaptive behavior with respect to its host spectrum. Finding new host-parasite associations may be important from a public health perspective, raising questions concerning the possible implication of these hosts as a source of human contamination by the tropical rat mite.

## Data availability statement

The datasets presented in this study can be found in online repositories. The names of the repository/repositories and accession number(s) can be found in the article/supplementary material.

## Ethics statement

Ethical review and approval was not required for the animal study because all diagnostic and therapeutic procedures were performed by licensed veterinarians in the course of routine veterinary health management. Written informed consent was obtained from the owners for the participation of their animals in this study. Written informed consent was obtained from the participant/patient(s) for the publication of this case report.

## Author contributions

MD and M-CC wrote the draft of the manuscript. FJ, JK, and M-CC performed the clinical examinations and microscope diagnoses. AG performed the molecular analysis. All authors contributed to writing the article, performed a critical review of the article, and approved the submitted version.
